# Sexual quality of life following a cancer diagnosis: a qualitative study

**DOI:** 10.1007/s00520-022-07459-8

**Published:** 2023-01-21

**Authors:** Lauren Haber, Andrew Allen, Karina T. Rune

**Affiliations:** School of Health and Behavioural Sciences, University of the Sunshine Coast, Sippy Downs, Australia

**Keywords:** Cancer, Sexual quality of life, Gender attachment, Cancer diagnosis

## Abstract

Sexual quality of life (SQoL) challenges are prevalent and persistent for people with a cancer diagnosis. Using a qualitative design, this study investigated the impact of a cancer diagnosis on SQoL in participants (*N* = 18) with a current or past cancer diagnosis of various sites and severity. Thematic analysis identified three themes: gender attachment, vulnerability, and growth vs. fixed mindset. The theme of gender attachment related to the impact of understanding SQoL based on heteronormative definitions of masculinity and femininity. The second theme of vulnerability suggested that pre-existing relational or personal challenges can amplify the impact of a cancer diagnosis on SQoL. The third theme of growth vs. fixed mindset explored how cognitive flexibility and optimism can support SQoL, while a rigid and pessimistic mindset can exacerbate SQoL challenges. The findings of this study build on the traditional medical understanding of SQoL and can inform SQoL support in cancer care settings.

**Supplementary Information:**

The online version contains supplementary material available at 10.1007/s00520-022-07459-8.

Sexual quality of life (SQoL) is an integral part of the human experience, relating to how we connect to ourselves and our intimate partners. Embodying the physical and emotional aspects of sex, intimacy, and body image [1, 2], SQoL is a lifelong consideration and is individualised in its significance and meaning. Some individuals value intimate touch and heartfelt conversation, while others place importance on intercourse and passion [3]. Sex and intimacy have been repeatedly identified as primary stressors for cancer patients across cancer types and phases of illness [4–6], with changes in their sexual relationship presenting as a significant challenge [7]. Although the primary goal for people with a cancer diagnosis may be survival, when focus shifts to the future, or to ensuring a fulfilling end of life period, the challenges and supports of SQoL become increasingly apparent [8–10].

## Challenging the biomedical perspective of SQoL

Most research on SQoL and cancer has adopted a biomedical perspective, exploring the physical effects of treatment and subsequent psychological impacts [7, 11]. Efforts to support SQoL are primarily at the request of the patient and may include psychosexual counselling and physical aides [9, 12]. However, recurrent distress associated with SQoL challenges reported by people with a cancer diagnosis suggests that there is more to this issue than is currently regarded. As SQoL is multifaceted, related challenges can encompass many aspects of a person’s life when it is not adequately supported. For example, poor SQoL has been linked to poor body image; fear of pain during sex; and shame surrounding sexual desires, which may impact confidence and lead to withdrawal of intimacy and relationship breakdowns [3, 13, 14]. Thus, understanding different aspects of SQoL is central to providing effective support for people with a cancer diagnosis.

### Frameworks and models of SQoL

Recent explorations of SQoL have aligned with the neo-theoretical framework of sexuality. Re-conceptualised by Cleary and Hegarty [15], this framework proposes that SQoL consists of sexual self-concept, including sexual self-schema, body image, and sexual esteem; sexual relationships, which includes communication and intimacy; and sexual function, including the sexual response cycle of desire, arousal, and orgasm. The neo-theoretical framework challenges the biomedical perspective often adopted by SQoL research and has led to a movement towards a more holistic understanding of sex and intimacy in people with a cancer diagnosis [16]. In conjunction with the neo-theoretical framework of sexuality, the pathway model of sexual adjustment proposed by Benoot et al. [7] considers the response of individuals with a cancer diagnosis and their intimate partners concerning the three categories of grief and mourning, restructuring, and sexual rehabilitation. Each of these pathways involves responding to the SQoL challenges that arise following a cancer diagnosis and account for diversity in response, due to factors such as cultural background and personal sexual preferences.

### Factors influencing SQoL

Many factors influence SQoL following a cancer diagnosis, including the perceptions of healthcare practitioners and their communication with patients, as well as the perceptions of patients and their communication with their partners. However, issues with sex and intimacy, whether physical or psychological, are often perceived as secondary to other health challenges [8] and influence both the practitioner’s willingness to offer support from the outset and the patient’s willingness to ask for support when challenges arise. This can be exacerbated by pre-existing relational or personal vulnerabilities, including relationship satisfaction and shame or embarrassment [17–19]. The reluctance of healthcare practitioners to discuss SQoL, due to factors such as lack of guidelines and uncertainty surrounding role relevance [8, 20, 21], further isolates people with a cancer diagnosis.

Patients’ perceptions of their identity concerning SQoL have been explored in previous research. White et al. [10] investigated sexuality following diagnoses, including gynaecological and anorectal cancer of various clinical stages, in interviews with 77 women aged 30 to over 70 years. Women that placed high importance on their vaginas in defining their sexuality reported poorer SQoL following treatment that altered their physical functioning or pleasure. Gurevich et al. [22] reported similar findings through interviews with 40 men with a testicular cancer diagnosis who were near various illness milestones. The men who placed high importance on the appearance and physical functioning of their penis, including fertility and erectile function, expressed poor SQoL both during and following treatment. Thus, the way people with a cancer diagnosis view and understand their SQoL is influential in the impact of the diagnosis on sex and intimacy. Through interviews with 32 women aged 35 to 77 years with metastatic breast cancer, McClelland [3] illustrated the different responses to physical, psychological, and social SQoL challenges in alignment with the neo-theoretical framework of sexuality and the pathway model of sexual adjustment. Four key themes emerged, namely unexpected embodied loss and mourning; silences; desires for others’ expertis; and worries about normalcy. This study highlights the current detrimental distinction between instrumental needs, such as information surrounding impacts to physical functioning and psychological well-being, reinforced by the over-medicalisation of SQoL in cancer care.

### Aim and rationale

Overall, the reviewed literature suggests the universality of the SQoL challenges faced by people with a cancer diagnosis. The issues raised, such as feelings of loss, preoccupation with partner perceptions, lack of communication, and a desire for normalcy, are not limited by factors such as age, gender, and disease site or severity. Using a qualitative research desing, the current study set out to understand the unique experiences, perceptions, and behaviours that impact SQoL following a cancer diagnosis. The overarching aim was to investigate SQoL following a cancer diagnosis. Specifically, the study explored themes relating to sex and intimacy in people with a cancer diagnosis.

## Method

### Participants and procedures

Ethical approval was granted by the University of the Sunshine Coast Human Ethics Committee. Participants were recruited via targeted social media advertisements, local community groups, cancer-relevant social media groups, and support group email lists. The final sample included 18 participants, at which point saturation was reached. A total of 31 accessed an online survey (hosted by Qualtrics), 10 were removed for not completing information beyond the demographic section, and three were removed due to requesting an option other than the online interview. Participants ranged from 26 to 81 years (*M* = 59.83, SD = 14.27). Seven identified as male and 11 as female. Participants had diagnoses of various types of cancer, with treatment and time since diagnosis also varying as outlined in Table 1. Participation was voluntary and open to any adult residing in Australia, with a current or past cancer diagnosis. Participants completed the survey online which took approximately 45 min.

**Table 1:** Summary of participants’ demographic information (*N* = 18)

Characteristic	*n*	%	Characteristic	*n*	%
Gender			Relationship status		
Male	7	38.9	Married	10	55.6
Female	11	61.1	Defacto	2	11.1
Age			Separated	5	27.8
18–30	1	5.6	Single	1	5.6
31–50	3	16.7	Relationship duration		
51–70	10	55.6	0–5 years	1	5.6
70 +	4	22.2	10–20 years	1	5.6
Sexual orientation			21 + years	10	55.6
Heterosexual	16	88.9	N/A	6	33.3
Bisexual	2	11.1	Time since diagnosis		
Gay	1	5.6	0–1 years	3	16.7
Cancer stage			2–5 years	9	50.0
Stage 1	2	11.1	6–10 years	3	16.7
Stage 2	1	5.6	11–20 years	2	11.1
Stage 3	1	5.6	20 + years	1	5.6
Stage 4	3	16.7	Cancer type		
In remission	4	22.2	Lung	1	5.6
Recovered	6	33.3	Ovarian	2	11.1
Unspecified	1	5.6	Breast	3	16.7
Current treatment			Papillary meningioma	1	5.6
Surgery	1	5.6	Thyroid	1	5.6
Chemotherapy	3	16.7	Phyllodes tumour	1	5.6
Hormone therapy	3	16.7	Bladder	1	5.6
Targeted therapy	1	5.6	Periampullary adenocarcinoma	1	5.6
None	6	33.3	Prostate	4	22.2
Unspecified	3	16.7	Glioblastoma	1	5.6
Other	1	5.6	Non-Hodgkins lymphoma	1	5.6
Past treatment			Squamous cell carcinoma	1	5.6
Surgery	8	44.4	Bowel	1	5.6
Chemotherapy	4	22.2	Tongue	1	5.6
Hormone therapy	2	11.1			
Radiation	2	11.1			
Unspecified	1	5.6			
Other	1	5.6			

### Materials

The study comprised an online survey, hosted via Qualtrics Survey Software, with demographic questions relating to personal and cancer-specific information and open-ended survey questions.

### Online interview

The online survey used 11 open-ended questions and prompts to guide responses designed to replicate a face-to-face or focus-group-style interview. The questions began broad (e.g. “Can you start by describing your relationship with your partner”) and gradually became more specific (e.g. “How was intimacy/sexual quality of life explored by health professionals during the cancer diagnosis?”). The questions were formulated in consultation with two clinical psychologists and based on previous research in oncology and SQoL in other chronic illnesses (e.g. [3, 23]. Revisions were made following a feedback session with an expert in the field of psycho-oncology to incorporate ethical considerations and ensure adequate sensitivity of the survey questions.

### Methodological integrity

The current study utilised a qualitative methodological approach to allow for a context-based understanding of the multi-dimensional factors that contribute to perceived changes in SQoL [24, 25]. The content of the online survey questions was primarily participant-led and consisting of open-ended questions. Although the predefinition of the interview questions may have restricted the scope of exploration [25], how the questions were answered, including length and depth of response, was decided by the participant. While the prompts provided some direction for the response, the content of the answers was defined by participants to ensure diversity relevant to the study population could be captured, as well as maintaining ethicality in the study delivery.

## Results

The data was analysed in accordance with Braun and Clarke’s [26] six-phase method of thematic analysis. The first phase involved familiarisation with the data, including reading the online survey responses, transferring them to NVivo for analysis, and making initial notes. These notes included highlighting items of interest such as references to body image and noting thoughts surrounding similarities and differences between the responses such as overall tone. The second phase involved generating initial codes by identifying terms that described the concepts expressed by participants, such as “second-hand sadness for SQoL changes”. Some codes reflected the language used by participants while others reflected themes and concepts within the dataset. A combination of direct transference of data to codes, as well as researcher interpretation, was used to generate initial codes, for example, “get on with life” and “multidimensionality of sexuality”. As the data was analysed, information of interest would either be added to an existing code, or if the data did not reflect any existing codes, a new code would be created. In the third phase, themes amongst the codes were identified. In this phase, meaning was attributed to the data in relation to the research question using the codes created. Similarities between the codes led to the generation of themes by grouping together codes that captured broader concepts. The overarching “story” weaving the themes together was also considered in this phase. Sixteen themes were identified in this stage (companionship, dependence, disconnect, fear, gender identity, growth mindset, healthcare issues, loss, neutral change, physical barriers, pre-existing issues, shared experience, stagnancy, survival, theory of mind, trauma of cancer). The fourth phase involved critically reviewing the identified themes and discerning whether the themes capture the data to describe a coherent story. Themes were questioned and modified or removed if there was insufficient data to support them, and redefined if they were deemed too broad or vague. During this stage, the themes were narrowed down to the final three (gender attachment, vulnerability, growth vs fixed mindset). Once the final themes were identified, the fifth phase involved creating definitions to describe the essence of each theme. This ensured uniqueness and relation to the research question. In the final phase, the report was produced in the form of the manuscript write-up [27].

Three themes were identified regarding the impact of a cancer diagnosis on SQoL (see Table 2). These themes were gender attachment, vulnerability, and growth vs. fixed mindset.

**Table 2:** Themes identified in relation to SQoL following a cancer diagnosis

Discourse and dimension	Example quote
Gender attachment	
SQoL is strongly linked to the perception of maintaining masculinity or femininity. People that have less attachment to their gender identity display less impacted SQoL	“It has left me feeling I have lost a lot of Manhood. I am no longer the complete package man.” (Participant 14, 81 years old, prostate cancer)
	“Society as a whole sexualises our bodies and treatments seem to be about preserving ‘perky breasts’ above all rather [*sic*] for our partners rather than what’s best for the patient.” (Participant 6, 57 years old, phyllodes tumour)
Vulnerability	
Pre-existing SQoL and relationship issues exacerbate the impact of diagnosis	“‘Back to normal’- not everyone’s life was perfect before cancer. Cancer can drag old skeletons out of cupboards—including unhappy sexual relations, failed expectations. Most people aren’t prepared for that.” (Participant 3, 75 years old, breast cancer)
	“I didn’t need to talk to anyone as we weren’t having sex anyway long before that.” (Participant 7, 71 years old, breast cancer)
Growth vs. fixed mindset	
People with more flexible thinking and adaptability experience less overall impact on SQoL than people that display more static and hopeless thinking	“Sex used to be super important and a way we stay close. Now we seem to have a good verbal companionship.” (Participant 2, 56 years old, ovarian cancer)
	“I’ve always enjoyed sex and it really pisses me off to have lost that aspect of life. I still haven’t gotten over it.” (Participant 8, 55 years old, bladder cancer)

### Gender attachment

The first theme identified was gender attachment. Four participants directly referenced gender, mentioning concepts such as manhood and femininity, and 10 referred to gendered body parts and acts of sex. The polarity of perspectives on SQoL and gender was illustrated through the contrasting comments of two participants. When answering the question: “How is intimacy related to your overall quality of life?”, one participant spoke about how the change in his role as the provider of his family following his diagnosis impacted his gender identity:

A return to the world we had pre-diagnosis would be a good start. Without those [sexuality and intimacy], on top of losing my career, I feel less of a man. My wife has kind of withdrawn from me, and I am feeling somewhat unloved, useless, impotent, a failure. (Participant 16, 66 years old, squamous cell carcinoma)

In contrast, in response to the question “Have changes to your body since the cancer diagnosis affected your intimacy/sexual quality of life?”, one participant described how changes to her self-view and understanding of her femininity supported her SQoL:

I am fine about my body. I have one breast. I’m not mutilated or less of a female person. Like wrinkles, my scar marks a passage and a challenge which I believe I have overcome. My partner has never had an issue either. It’d be a sad thing if your person or spirit was limited to one mammary gland. (Participant 6, 57 years old, phyllodes tumour)

Generally, the participants who conceptualised SQoL in relation to their gender role and identity used more negative language than those focused on non-gendered facets of SQoL such as intimacy and relationship quality when describing the impact of their cancer diagnosis.

### Vulnerability

The second theme that emerged was vulnerability. Participants that spoke about pre-existing relational or personal SQoL challenges (e.g. poor communication, insecurities, prior decline in sexual activity) reported greater negative changes in their SQoL than those that spoke about the strength of their relationship and character. Relationship tension and diminishing sex life prior to cancer diagnosis were perceived to be the stressors, with the cancer diagnosis and subsequent increase in stress the breaking point for SQoL. One participant spoke of his disappointment in the state of his SQoL, placing the blame on his partner’s belief that they are too old for sex:

I just think that sexual acts are forever, not when a partner thinks that it’s finished because they are 66. (Participant 15, 69 years old, non-Hodgkins lymphoma and tongue cancer)

Another participant reiterated this frustration with differing perspectives on SQoL in his relationship and the challenge this creates when navigating SQoL following a cancer diagnosis:

Conversations of an intimate nature are awkward to say the least and rarely take place… I am aware (as is my wife) that there are many health benefits derived from an active sex life, but the inertia required to overcome this is just too great at the moment. (Participant 16, 66 years old, squamous cell carcinoma)

The way relationship quality, including shared beliefs and communication, can support SQoL following a cancer diagnosis was expressed. This demonstrates that the theme of vulnerability can also apply in reverse. In describing his relationship with his “very supportive wife”, a participant said:

Intimacy and sexuality played and continues to play a very important part of our relationship. That importance has not changed since diagnosis. Mutual satisfaction and openness in conversation and actions are important to both of us…Sexual quality of life to me means having an enjoyable and fulfilling intimate relationship. That has not altered since diagnosis but perhaps brought to [*sic*] more into focus, not knowing the future. (Participant 17, 67 years old, prostate cancer)

Participants that described their relationship as distant and strained reported poorer SQoL than those that expressed the supportiveness of their partner and strength of their relationship.

### Growth vs. fixed mindset

The third theme identified was growth vs. fixed mindset. Through their responses, participants could be divided into those that displayed a growth or fixed mindset approach to SQoL following a cancer diagnosis. Those categorised as displaying a growth mindset spoke about SQoL as fluid, expressing that it was something able to be renegotiated and improved.

Conversations centred around if there was no sex/intimacy we have to accept it. Yes. It [was] as we were warned. The desires have not changed but unable to perform the actions. Take action if available to restore the capability… We have accepted the situation as it is, we decided we would take pills to change the situation. (Participant 18, 81 years old, prostate cancer)

In contrast, those who could be categorised as displaying a fixed mindset spoke about SQoL as lost following the diagnosis expressed feelings such as anger, fear, grief, and regret. Participant 4 (26 years old, papillary meningioma) spoke about fear as a barrier to intimacy, mentioning that her relationship breakdown was “inevitable”. Participant 4 detailed how her SQoL was dominated by fear, saying that she is “more fearful of intimacy generally because of the ‘baggage’ of cancer” and the physical impacts of cancer treatment are “insurmountable”. Participants in the fixed mindset category often used negative and self-degrading language:

During my illness, my fitness deteriorated, and I lost the tone. Was on steroids for a while and was bloated. Eventually weight dropped during chemo and radiation. I was bald, skinny, loose skin. So what I saw in the mirror was disgusting and my partner did not see me naked when I became independent. (Participant 13, 59 years old, glioblastoma)

Within this group, there was a commonality in the perception of SQoL as something that could not be recovered or repaired, resigning to the belief that it was lost and often using negative absolutes to describe changes in sex and intimacy. When responding to the question “What has challenged your intimacy/sexual quality of life?”, Participant 10 (50 years old, breast cancer) stated: “The challenge is accepting you won’t have any again.”. Similarly, when answering “What advice would you give to couples going through the same experience to support their intimacy/sexual quality of life”, Participant 8 (55 years old, bladder cancer) expressed: “Wondering if I’ll ever find a partner. Wondering why anyone would be interested”. Despite significant contrast in the experiences of those in a long-term relationship compared to those who were in new relationships or single, this defeatism was consistent amongst participants that reported poorer SQoL than those that responded with optimism.

## Discussion

This study investigated the impact of a cancer diagnosis on SQoL, including sex, intimacy, and body image. Three key themes were identified through the interviews, namely gender attachment, vulnerability, and growth vs. fixed mindset. Participants who appeared to have less attachment to their gender and focused less on aspects of their SQoL related to heteronormative definitions of masculinity and femininity (e.g. maintaining an erection, having breasts, ejaculation) spoke more positively about their SQoL. This aligns with the dimension of sexual self-concept in the neo-theoretical framework of sexuality [15]. Having a sexual self-concept grounded in rigid definitions of masculinity and femininity can impact SQoL through body image and sexual self-esteem as people may perceive their sexuality to be impacted based on deviation from male and female societal norms [16, 28].

The findings of this study corroborate previous research suggesting that undergoing radical surgery such as mastectomy or prostatectomy can have a negative impact on body image and SQoL generally [29, 30]. This was indicated through participants’ references to regret surrounding surgery that impacted their physical appearance and functioning. For example, Participant 11 (63 years old, prostate cancer) said “[I] wished I never had my prostate removed”. The concept of loss in the context of gender identity was also explored by McClelland [3] in questioning whether women “missing their boobs” (p.424) was attributed to their own sexual self-concept or cultural norms and perceived partner expectations. In this way, gender attachment can be seen to interplay with growth vs. fixed mindset, as described in the current study.

The participants in the current study that reported feelings of loss and grief for their SQoL often referenced their changing gender identity and having lost aspects of this, such as having large breasts or the ability to maintain an erection. This corroborate past research, with White et al. [10] reporting that physical changes often result in feeling a loss of femininity. Lee et al. [31] reported similar findings in males, with erectile function and ejaculation ability influencing feelings of masculinity. In contrast, some participants in the current study actively rejected conforming to societal gender norms and the concept of rigid gender identity. Similar findings have been reported by Gurevich et al. [22] who reported that while navigating physical and functional changes, some men choose to challenge their previous understanding of masculinity, turning away from the biomedical perspective of sex and towards definitions that incorporate the psychological and social aspects of SQoL.

The effects of societal norms and expectations of SQoL within the sexuality- and gender-diverse community are points of interest. In the current study, the only gay participant reported that the cancer diagnosis had negatively impacted his SQoL due to the expectations surrounding erection and ejaculation in the gay community. Although inferences cannot be drawn from one participant’s experience alone, the sentiment is echoed in existing literature. For example, when comparing gay and heterosexual men with prostate cancer, Hart et al. [32] reported that gay men had poorer SQoL, including greater disease fear. In contrast, Ussher et al. [14] proposed a counter-narrative of stable SQoL in lesbian and poly-sexual women with a cancer diagnosis, possibly due to greater body change acceptance and lower importance of penetrative sex. As such, based on previous research and the results of the current study, it appears that lack of gender attachment may be a protective factor for SQoL. However, further investigation into gender- and sexuality-diverse populations is needed to investigate the importance placed on definitions of gender by society apparent in both heteronormative and sexuality- and gender-diverse populations when navigating SQoL following a cancer diagnosis.

Pre-existing vulnerabilities were identified as another key factor influencing the impact of a cancer diagnosis on SQoL. Participant 3 summarised this theme by saying that “not everyone’s life was perfect before cancer. Cancer can drag old skeletons out of cupboards”. Relationship vulnerabilities such as tension, reduced intimacy, poor communication, and overall relationship quality prior to diagnosis may exacerbate SQoL challenges and prevent positive changes as described in the pathway model of sexual adjustment. This is corroborated by Walker and Robinson [33], suggesting that while a cancer diagnosis is likely to incur SQoL challenges, couples that exhibit indicators of high relationship quality such as open communication and a desire for mutual pleasure are likely to have a more positive experience when navigating sex and intimacy.

Pre-existing personal vulnerabilities and insecurities, including body image distress, were also seen in this study to influence SQoL outcomes, with participants that reported issues with their body image or general insecurities about sex and intimacy speaking more negatively about their SQoL. The relationship between existing body image distress and poor SQoL is corroborated by Ljungman et al. [34], with findings identifying negative body image as a significant predictor of poor SQoL. Reese et al. [13] extended on this, proposing that women are more susceptible to SQoL challenges due to higher baseline body image distress than men. These findings emphasise the importance of early assessment of patient vulnerabilities, in particular body image distress.

The third theme identified, growth vs. fixed mindset, is arguably the most important in shaping SQoL support following a cancer diagnosis. Through interpretation of the underlying tone of the interviews, it became apparent that participants who spoke about their SQoL through a lens of optimism, hope, and flexibility reported more positive SQoL outcomes than those that spoke about SQoL with pessimism, despair, and stagnancy. These findings are supported by the pathway model of sexual adjustment, with the chosen response of both the individual and couple following a cancer diagnosis impacting SQoL outcomes. Corroborated by Hawkins et al. [35], responding to SQoL challenges with feelings of guilt and loss can negatively influence intimate relationships. In choosing to view SQoL challenges, including changes to sex and intimacy, as a part of the disease process and employing strategies to adapt and rehabilitate, the impacts of a cancer diagnosis are not experienced as strongly [33, 36]. This aligns with the findings of this study, with the participants that discussed employing adjustment strategies such as seeking psychological support or using aids (e.g. pharmaceuticals, sex toys) reporting less impact on their SQoL. The perception of SQoL as fixed may prevent help-seeking behaviours in people with a cancer diagnosis and contribute to a detrimental decline in sex, intimacy, and relationship engagement. In contrast, perceiving SQoL as fluid and adaptable may support patients in navigating SQoL changes following a cancer diagnosis. However, this modality of support is contingent on healthcare practitioners operating within a biopsychosocial framework [3, 37] and encourages patients to consider the social, psychological, and physical impacts of a cancer diagnosis on SQoL when undergoing cancer treatment.

This study has several limitations. First, the predefinition of the qualitative interview questions may have restricted the scope of exploration [25], resulting in biased data collection. However, to reduce distress, due to the potentially sensitive and highly personal content of the interviews, and participant reactivity [38], the interview questions were framed as open-ended to allow participants to divulge as little or as much detail as they desired. Second, COVID-19 restrictions impacted data collection. While the study design initially involved focus-group-style interviews, restrictions impacting face-to-face contact required the questions to be reformatted to suit online delivery. The decision to use a written-response survey was based on the richness of data achieved in studies of similar design (e.g. [39–41]) and endorsement by Braun et al. [42]. This also resulted in an increased need for interpretation of responses by the research team, as no clarification on tone, inference, or meaning could be sought from participants as would occur in a face-to-face interview. The research team took measures to prevent subjectivity including individually reviewing the responses and consultation with experts to review findings. Third, the study sample did include participants identifying as gay and bisexual, as well as various relationship statuses, ages, and disease site and severity. However, the qualitative methodology resulted in a sample heavily weighted towards an older, heterosexual population in long-term relationships. The topic of investigation warrants maximum population diversity, including participants of other sexual orientations and gender identities. Future research should aim to increase population diversity to both prevent data bias and better understand the potential influence of factors such as social group norms and gender differences on SQoL. While a heterogenous sample may seem to dilute the applicability of the findings, this was the first study of its kind to explore the impact of a cancer diagnosis on SQoL using the neo-theoretical framework of sexuality. Thus, by working to identify the factors that influence SQoL in people with a cancer diagnosis, there is hope to understand any variation in impact across homogenous samples.

The present study has implications for understanding and addressing SQoL challenges in people with a cancer diagnosis. Considering the findings in conjunction with previous literature, communication surrounding the impact of cancer on SQoL should be addressed at the outset of diagnosis and addressed in a manner reflective of both the prevalence and uniqueness of SQoL challenges. Additionally, to appropriately support individuals with a cancer diagnosis, healthcare provider advice should be based on understanding the nuance of SQoL and the uniqueness of navigating sex and intimacy while simultaneously navigating a cancer diagnosis. The three key themes identified highlight the importance of considering the biopsychosocial impacts of a cancer diagnosis on SQoL at the outset and imply that SQoL can be supported by addressing the existing vulnerabilities in patients’ cognition, self-image, schemas, and support systems.

In conclusion, thematic analysis revealed gender attachment, vulnerability, and growth vs. fixed mindset as key themes in understanding the impact of a cancer diagnosis on SQoL. The findings of this study show that SQoL is a pervasive and multifaceted challenge faced by people with a cancer diagnosis indiscriminate of demography. Although previous research suggests particular cancer sites attract greater SQoL challenges, this study does not support this, instead highlighting the pervasiveness of SQoL challenges. Additional research is recommended to widen the scope of investigation and redefine SQoL in the context of a cancer diagnosis, as it has significant implications for patients and their intimate partners.

## Supplementary Information

Below is the link to the electronic supplementary material.

Supplementary file1 (DOCX 770 KB)
