# Supplementary material for: Sexual quality of life following a cancer diagnosis: a qualitative study
Source: Support Care Cancer. 2023 Jan 21;31(2):125. doi: 10.1007/s00520-022-07459-8 (PMC9860240; doi:10.1007/s00520-022-07459-8)

**Supplementary Materials**

**Screening questions**

1. Please indicate your age
2. Are you currently living in Australia?
3. Are you a current cancer patient or survivor of cancer?

**Demographic questions**

1. What gender do you identify as?
2. How would you describe your sexuality?
3. How many years have you been in your current relationship?
4. When were you diagnosed with cancer?
5. Please specify what type(s) of cancer you have/had
6. Please specify what stage your cancer is at
7. If currently undergoing cancer treatment, please specify what type of treatment(s)
8. If you received cancer treatment in the past, please specify what type of treatment(s)

**Interview questions**

1. Can you tell us a little about your relationship with your partner
2. Can you tell us how cancer has impacted your relationship with your partner

Probe: How has cancer impacted intimacy with your partner

1. How is intimacy related to your overall QoL?

Probe: Does intimacy impact your overall QoL?

Probe: Has this importance changed since your cancer diagnosis?

Probe: How important is sexuality to your QoL?

Probe: Has this importance changed since your cancer diagnosis?

Probe: What aspects of sexuality and intimacy are most important to you now?

1. What does sexual quality of life mean to you?

Probe: How would you define sexual quality of life?

Probe: How has the meaning of sexual quality of life changed since your cancer diagnosis?

Probe: How does sex and intimacy relate to sexual quality of life for you?

Probe: How does sex and intimacy relate to sexual quality of life for your partner?

1. How have you had to renegotiate intimacy/sexual quality of life with your partner since the cancer diagnosis?

Probe: Has your cancer diagnosis affected your intimacy/sexual quality of life in the way you thought it would?

Probe: What would you change about your intimacy/sexual quality of life, if anything?

1. What has supported your intimacy/sexual quality of life?

Probe: How have you reacted?

Probe: How have you coped?

Probe: How has your partner reacted?

Probe: How has your partner coped?

1. What has challenged your intimacy/sexual quality of life?

Probe: How have you reacted?

Probe: How have you coped?

Probe: How has your partner reacted?

Probe: How has your partner coped?

1. Have changes to your body affected your intimacy/sexual quality of life?

Probe: Has the way you view your body changed since the cancer diagnosis?

Probe: How has your body image impacted your intimacy/sexual quality of life since the cancer diagnosis?

1. How was intimacy/sexual quality of life explored by health professionals during the cancer diagnosis?

Probe: Is there anything you wish you had been asked/discussed by health professionals regarding your intimacy/sexual quality of life?

Probe: What conversations have you and your partner had, if any, regarding sex and intimacy or sexual quality of life?

1. What advice would you give to couples going through the same experience to support their intimacy/sexual quality of life?
2. Is there anything we haven’t spoken about regarding intimacy/sexual quality of life in the context of managing cancer that you believe needs to be mentioned?

**Coding Trees**


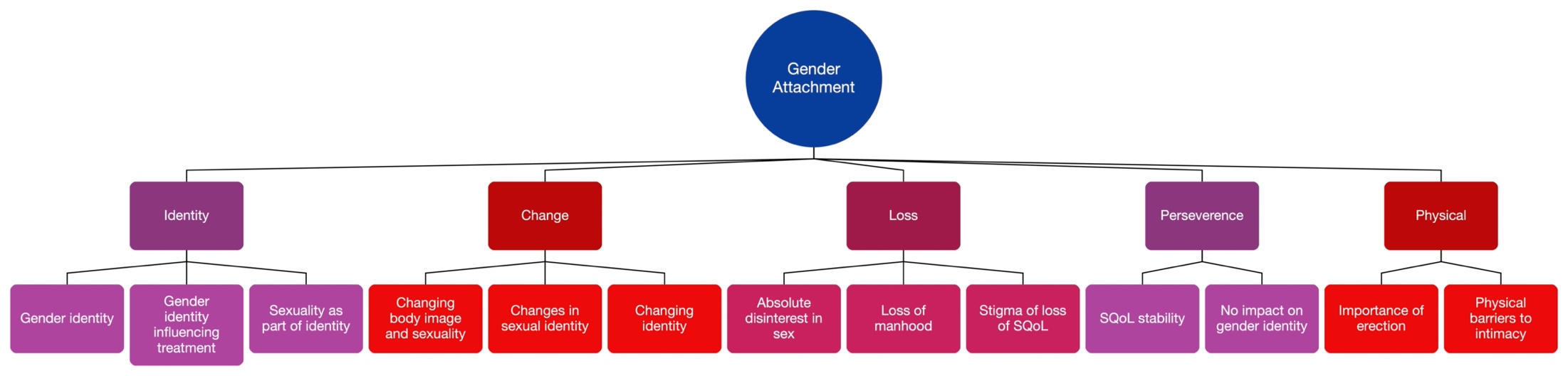


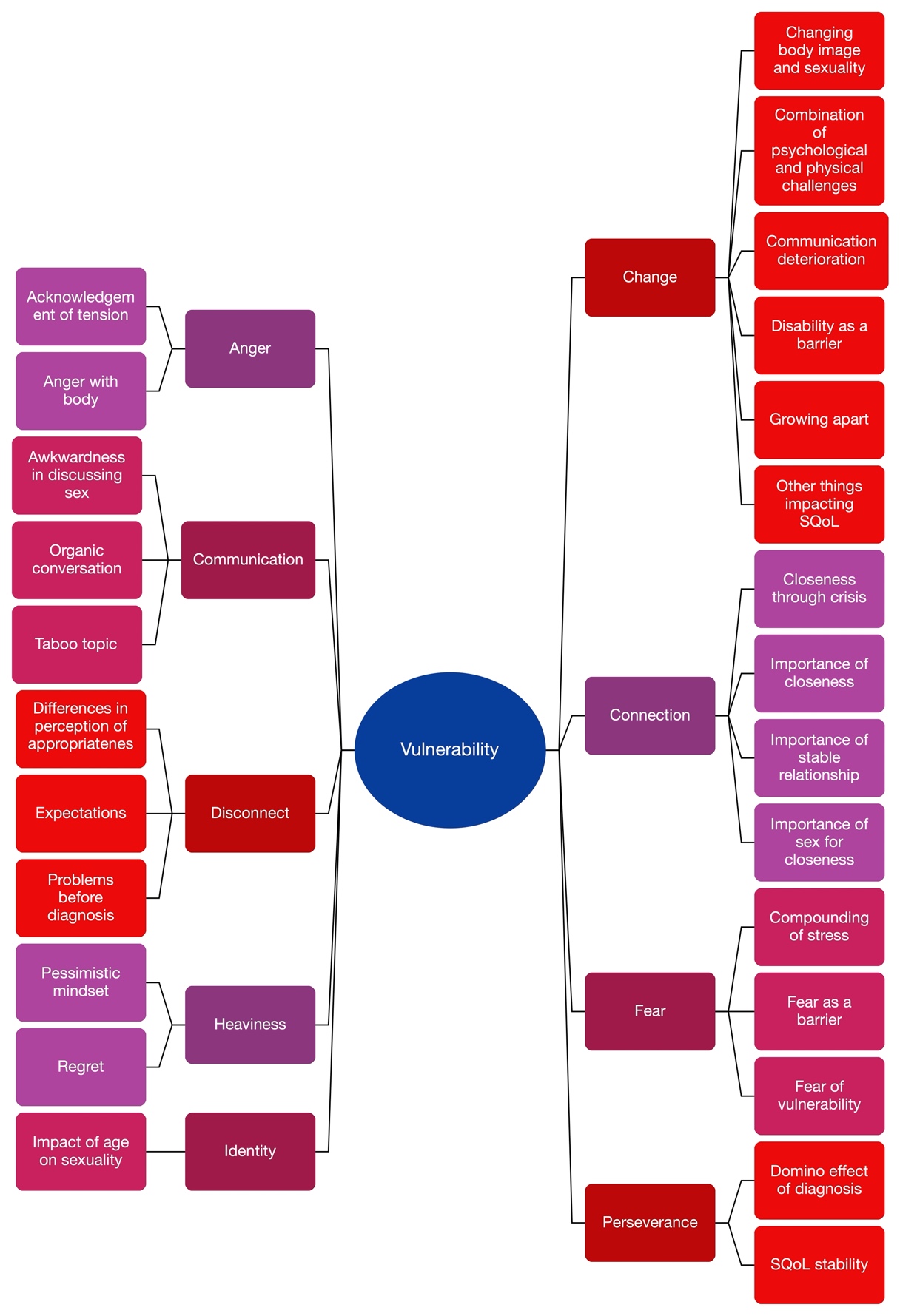


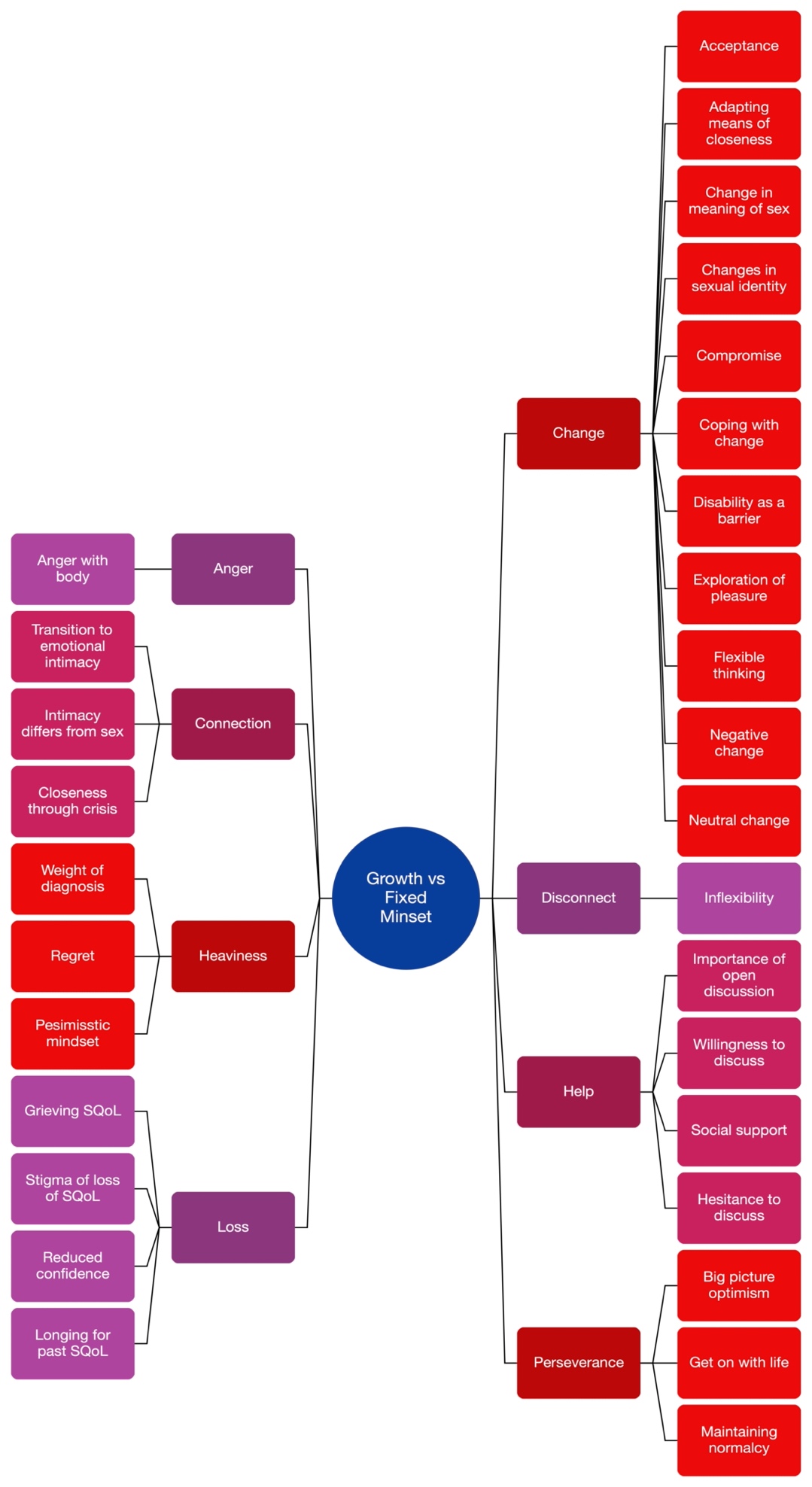

Supplement: Supplementary file 1 — Supplementary file1 (DOCX 770 KB) [file 520_2022_7459_MOESM1_ESM.docx]
